# Fatigue and neuropsychiatric symptoms in Parkinson’s disease: a narrative review

**DOI:** 10.3389/fneur.2025.1670644

**Published:** 2025-10-24

**Authors:** Lidia Bojtos, Jon Rodríguez-Antigüedad, Javier Pagonabarraga, Saül Martínez-Horta, Jaime Kulisevsky

**Affiliations:** ^1^Medicine Department, Universitat Autònoma de Barcelona (UAB), Barcelona, Spain; ^2^Movement Disorders Unit, Neurology Department, Hospital de la Santa Creu i Sant Pau, Barcelona, Spain; ^3^Institut d’Investigacions Biomèdiques-Sant Pau (IIB-Sant Pau), Barcelona, Spain; ^4^Centro de Investigación Biomédica en Red-Enfermedades Neurodegenerativas (CIBERNED), Madrid, Spain

**Keywords:** fatigue, neuropsychiatric symptoms, Parkinson’s disease, symptom overlap, treatment

## Abstract

Fatigue in Parkinson’s disease (PD) is a highly prevalent and disabling non-motor symptom, often manifesting early and worsening over the disease course. Despite its significant impact on quality of life, fatigue remains underrecognized and poorly managed in clinical practice. It is a complex, multidimensional syndrome encompassing cognitive, emotional, and physical components. Its pathophysiology is multifactorial, involving disrupted dopaminergic, serotonergic, and glutamatergic signaling across fronto-striatal and limbic circuits. Fatigue frequently overlaps with neuropsychiatric symptoms such as apathy, depression, anxiety, and cognitive impairment, complicating both diagnosis and treatment. Although these conditions are clinically distinct, they share overlapping neural substrates and may influence each other’s presentation and severity. Currently available therapeutic options for PD-related fatigue are limited, with rasagiline considered only “possibly useful,” and most other pharmacologic and non-pharmacological strategies lacking rigorous evidence. This narrative review is based on a non-systematic PubMed literature search of peer-reviewed articles in English up to April 2025, with additional relevant studies identified through reference lists. It examines the clinical and neurobiological intersections between fatigue and neuropsychiatric symptoms in PD, highlighting key diagnostic challenges, treatment limitations, and future directions. Standardizing terminology, dissecting fatigue from overlapping neuropsychiatric symptoms, identifying reliable biomarkers, and conducting well-designed, mechanism-based clinical trials are essential next steps to redefine fatigue as a measurable and treatable symptom in PD.

## 1 Introduction

Parkinson’s disease (PD) is a neurodegenerative condition manifesting with both motor and non-motor symptoms (NMS) as a result of the widespread degeneration not only of the nigrostriatal dopaminergic system, but also of non-dopaminergic systems extending beyond the nigrostriatal pathway (1). Since 1993, fatigue has been recognized as an independent and frequent NMS in PD (2). Pathological fatigue is a syndrome characterized by a sense of exhaustion that cannot be explained by medications or other medical (cardiac disease, orthostatic hypotension, anemia, thyroid disorders) or psychiatric condition, and is severe enough to interfere with normal activities (3).

Fatigue in PD is highly prevalent, affecting 50% of patients (4). It can manifest from the prodromal stages, and typically persists and worsens over the course of the disease (4–7). Fatigue is a significant symptom not only due to its prevalence but also because of its substantial impact on quality of life, being recognized as one of the most disabling symptoms of the disease (4).

While most research has focused on its physical aspect, fatigue is a multidimensional symptom that also includes emotional and cognitive components (3, 8). Importantly, Kluger et al. proposed a unified taxonomy distinguishing between *fatigue*, defined as a subjective perception of weariness, lack of energy, or disproportionate effort, and *fatigability*, an objectively measurable decline in performance relative to a reference over time (9). This framework emphasizes that fatigue and fatigability are dissociable phenomena that can occur independently, a distinction that has proven critical in PD, where subjective complaints of fatigue often do not correlate with objective motor performance decrements. Recognizing this distinction early in the disease characterization is essential to disentangle underlying mechanisms and guide targeted interventions.

The pathophysiology remains unclear but is considered a primary symptom of PD, resulting from the neurodegenerative process (10). Despite the similarities between PD-associated fatigue and fatigue in other neurological conditions such as multiple sclerosis or cancer, it is believed to involve disease-specific mechanisms. As with other NMS, various mechanisms have been proposed as potential contributors to fatigue in PD, including dopaminergic and/or serotonergic dysfunction, disrupted connections between the basal ganglia and prefrontal cortex, and pro-inflammatory factors (11–14). These mechanisms are thought to create an imbalance between the perceived effort and the reward gained from that effort (10).

Research on fatigue has been considerably limited by the inherent ambiguity of the term itself, as well as by the heterogeneity of the assessment methods used. In the context of PD-associated fatigue, an additional challenge lies in the significant overlap with other independent NMS, mainly neuropsychiatric, both in terms of clinical manifestations and underlying pathophysiology. These complexities have hindered progress in its understanding and contributed to a lack of clinical trials, thereby limiting the development of effective treatments (15–18).

The main objective of this narrative review is to explore the differences and similarities between fatigue and neuropsychiatric symptoms in PD, from both clinical and pathophysiological perspectives. Given the frequency, impact, and specific treatment approaches for neuropsychiatric symptoms in PD, accurately identifying whether fatigue or other neuropsychiatric manifestations represent the predominant component of a patient’s symptomatology is essential for guiding appropriate interventions.

## 2 Search strategy

We conducted a non-systematic literature search of peer-reviewed articles published in English up to April 1, 2025. The search was performed using the PubMed database. The terms used for this search included: (“Parkinson*” AND (“fatig*”)), combined with (“neuropsychiatric” OR “psychiatric”), “apath*,” “depressi*,” “anxiety,” “cogniti*,” (“psychosis” OR “hallucinations” OR “delusion”), and (“impulse control OR impuls*”). Additional studies were identified through references in the included manuscripts, and those deemed relevant were selected.

## 3 Main challenges in PD-associated fatigue

The three main challenges of PD-associated fatigue, in both clinical and research settings, are the terminology used to define fatigue, the assessment methods applied, and its frequent co-occurrence with other NMS.

First, the term *fatigue* is highly ambiguous, both for patients and in research. On the one hand, the general population may describe actual fatigue in a wide variety of ways (e.g., tiredness, heaviness, feeling overwhelmed, blunted affect, or lack of motivation), and it can also be semantically and/or symptomatically confused with other conditions (e.g., normal physical exhaustion, common cold, dyspnea, depression, apathy, or sleepiness) (8). On the other hand, researchers have used the same term to refer to different aspects of fatigue (e.g., *central fatigue* has been used to describe subjective fatigue, fatigue originating in the central nervous system, or cognitive fatigue; *mental fatigue* has also been used as a synonymous of fatigue) (9, 19). For this reason, recent efforts have focused on standardizing the terminology and establishing a more specific definition (Table 1) (9, 15, 20).

**Table 1:** Clinical diagnostic criteria for PD-associated fatigue (15, 20).

Patients must report significantly diminished energy levels or increased perceptions of effort that are disproportionate to attempted activities or general activity level. Symptoms must be present for most of the day, every day, or nearly every day during the previous month. In addition, patients must have four or more of the symptoms from Section A and meet criteria in Sections B, C, and D.
A. Symptoms Symptoms may be induced by routine activities of daily living Symptoms may occur with little or no exertion Symptoms limit the type, intensity, or duration of activities performed by the patient Symptoms are not reliably relieved by rest or may require prolonged periods of rest Symptoms may be brought on by cognitive tasks or situations requiring sustained attention including social interactions Patients avoid rigorous activities because of fear of experiencing worsening of symptoms Mild to moderate exertion may induce a worsening of symptoms lasting hours to days Symptoms have a predictable diurnal pattern regardless of activities performed (e.g., worsening in the afternoon) Symptoms are unpredictable and may have a sudden onset
B. The patient experiences clinically significant distress or impairment in social, occupational, or other important areas of function as a result of fatigue
C. There is evidence from the history and physical examination suggesting fatigue is a consequence of Parkinson’s disease
D. The symptoms are not primarily a consequence of comorbid psychiatric disorders (e.g., depression), sleep disorders (e.g., obstructive sleep apnea), or medical conditions (e.g., anemia, congestive heart failure)

Second, the lack of terminological clarity is a major limitation to the development of reliable scales for effectively using fatigue as an outcome in clinical trials. While objective fatigue can be assessed through sustained or repeated motor or cognitive tasks by measuring performance changes, subjective fatigue remains more difficult to quantify and often does not correlate with objective measures (3). The Movement Disorders Society (MDS) Task Force published a comprehensive review in 2010 on scales designed to assess subjective fatigue (21). For screening purposes, the Fatigue Severity Scale (FSS) and the Parkinson Fatigue Scale (PFS) are recommended, whereas the FSS and the Multidimensional Fatigue Inventory (MFI) are recommended for evaluating severity (21). Some of these scales, such as the MFI, successfully capture the multidimensional nature of fatigue, including its cognitive and emotional components, such as reduced motivation. Beyond fatigue-specific scales, fatigue can also be indirectly captured in broader instruments such as the Non-Motor Symptoms Scale (NMSS, sleep/fatigue domain) and the MDS-UPDRS (Part I, item 1.13).

Finally, fatigue in PD often overlaps with other common NMS, particularly neuropsychiatric symptoms such as apathy, depression, anxiety, and cognitive decline (11, 22). Since each is thought to involve distinct pathophysiological mechanisms, including specific neural networks and neurotransmitter systems, accurate differentiation is essential for a tailored treatment approach. While rasagiline is currently the only treatment considered *possibly useful* by the MDS-Evidence Based Medicine for PD-associated fatigue, cognitive impairment is managed with acetylcholinesterase inhibitors, apathy with dopaminergic agents or acetylcholinesterase inhibitors, and anxiety and depression are commonly treated with antidepressants (18, 23). A comprehensive neuropsychological assessment may help better characterize fatigue and co-occurring emotional and cognitive symptoms, thereby guiding appropriate management strategies.

## 4 Pathophysiological mechanisms of fatigue

Understanding the biological basis of fatigue in PD is essential, both to disentangle it from overlapping neuropsychiatric symptoms and to identify candidate biomarkers for clinical use. Converging evidence from neuroimaging, electrophysiological, and inflammatory studies implicates fronto-striatal and limbic dysfunction, neurotransmitter imbalances, and immune-related processes as key contributors to fatigue pathophysiology.

Fatigue has been attributed to a failure to integrate limbic input with motor functions within the basal ganglia due to the disfunction of striato-thalamo-cortical circuit (10). The dysfunction in linking motivation to task execution may explain why patients with fatigue report a higher perceived effort, even when they are physically capable of completing the task. Functional neuroimaging studies support this model. Altered metabolism and disrupted functional integrity in the prefrontal cortex have been demonstrated in fatigued patients (24, 25). Further studies using SPECT with 99mTc-HMPAO, FDG-PET, and resting-state fMRI have consistently reported abnormalities involving the prefrontal cortex, striatum, and cingulate cortex, along with additional alterations in the insular and parietal regions (25–28). In line with these imaging findings, a recent study using quantitative electroencephalography (qEEG) highlighted fronto-striatal dysfunction in PD patients with fatigue, demonstrating reduced network efficiency in frontal regions, while increased efficiency in temporal regions may reflect compensatory mechanisms (29).

At the structural level, findings are less consistent. A few isolated studies have reported associations between fatigue and reduced gray matter volume in the dorsal striatum, as well as alterations in white matter tracts (30, 31). Among the limited available data, some studies have identified associations between fatigue and cortical atrophy, whereas others have found no significant cortical structural changes (24, 31, 32).

Evidence from molecular imaging further implicates neurotransmitter dysfunction. Reduced serotonin transporter uptake in the caudate, putamen, ventral striatum, and thalamus has been demonstrated with [^11^C]-DASB PET (14). Fatigue has also been linked to nigrostriatal dopaminergic denervation, suggested by reduced [^11^C]-DTBZ uptake, while diminished [^18^F]-DOPA uptake in the insular cortex points to the involvement of extrastriatal dopaminergic systems (14, 33). More recent studies indicate that fatigue may also arise from disruptions in broader neurotransmitter-enriched networks. Widespread reductions in noradrenaline-enriched connectivity within the sensorimotor, salience, and default mode networks, together with decreased glutamate-enriched connectivity in the supplementary motor area, have been observed, both showing inverse correlations with fatigue severity (34).

Beyond neurotransmitter dysfunction, accumulating evidence points to an inflammatory contribution to PD-related fatigue. It has been consistently linked to elevated levels of peripheral inflammatory markers, including interleukin-1 beta (IL-1β), interleukin-6 (IL-6), interleukin-18 (IL-18), soluble interleukin-2 receptor (sIL-2R), tumor necrosis factor alpha (TNF-α), high-sensitivity C-reactive protein (hs-CRP), neutrophil-to-lymphocyte ratio (NLR), and red blood cell distribution width (RDW) (35–40). In addition, higher cerebrospinal fluid (CSF) levels of C-reactive protein (CRP) and monocyte chemotactic protein-1 (MCP-1) have been associated with more severe fatigue, depression, and cognitive impairment in PD, supporting the role of central inflammation in these overlapping symptoms (12). Alongside elevated pro-inflammatory markers, fatigue in PD is also associated with increased levels of neuronal damage markers, including neuron-specific enolase (NSE), S100 calcium-binding protein B (S100B), phosphorylated tau217 (pTau217), and glial fibrillary acidic protein (GFAP), underscoring the interplay between peripheral immune-inflammatory activation and damage to glial projections and neurofilaments in its pathophysiology (41). Additionally, a recent study indicated that higher baseline neurofilament light chain (NfL) levels were associated with both persistent and new-onset fatigue, suggesting that NfL may serve as a biomarker for predicting fatigue progression (42).

## 5 Fatigue and apathy

Apathy is a common NMS associated with PD. It is mainly defined as a motivation disorder and is operationalized as diminished behavior, emotion, and cognition in goal-oriented tasks (43). Its frequency ranges from 17 to 54% depending on the population characteristics and assessment procedures (44–46). Apathy is known to contribute significantly to caregiver burden and has negative implications for treatment and long-term outcome (47–49).

While fatigue and apathy are two distinct syndromes, they are closely related and frequently co-occur in PD. Due to the symptomatic overlap, current diagnostic frameworks and rating instruments frequently conflate apathy with fatigue, leading to inaccurate diagnoses and sub-optimal treatment strategies. PD patients with fatigue exhibit moderately higher levels of apathy, and conversely, fatigue has been described significantly more prevalent in untreated, early-stage apathetic PD patients compared to non-apathetic ones (4, 50).

When analyzing the individual domains of apathy, cognitive inertia and auto-activation deficit correlate the strongest with fatigue: patients with greater cognitive and behavioral apathy tend to feel the most fatigued (51). In line with these findings, in early, untreated PD patients, cognitive apathy has been identified as an independent predictor of distressing fatigue (52). Part of this association may arise from conceptual overlap between the two constructs and the potential for misdiagnosis: diminished goal-directed behavior is characterized by lack of effort or energy to perform everyday activities, which is also considered a clinical consequence of physical fatigue (53). Yet other apathy features (such as dependency on prompts from others to organize daily activities, disinterest in learning new things, and indifference toward personal problems) are likewise more common in fatigued patients, suggesting that this association is not merely due to symptom overlap or misdiagnosis, but may reflect shared neural correlates involving the basal ganglia–dorsolateral prefrontal cortex loop (51).

Similarly to patients with fatigue, disruptions of prefrontal cortex–basal ganglia circuits has also been observed in apathetic patients (54). Lesions in these pathways are associated with emotional apathy, characterized by a reduced ability to integrate affective signals into ongoing and future behavior (55). Such dysfunction is already detectable in the early stages of PD, where higher baseline levels of emotional apathy have been liked to greater fatigue severity over time (56). These findings suggest that both symptoms may arise from shared impairments in emotional-affective processing, likely involving overlapping pathophysiological mechanisms within the limbic regions of the basal ganglia (56).

Neuroimaging studies further highlight shared alterations in neurotransmitter systems, with both fatigue and apathy linked to dysfunction in dopaminergic and serotonergic circuits. Apathy has been particularly linked to mesolimbic dopaminergic denervation (57–60). Serotonergic dysfunction within the corticolimbic-basal ganglia circuit, especially in the early stages of the disease, has also been observed (61, 62). While striatal dopaminergic denervation may primarily contribute to fatigue in early-stage PD, serotonergic dysfunction may increasingly influence fatigue as the disease progresses (33). Conversely, serotonergic lesions appear to play a crucial role in apathy during early-stage PD, with extrastriatal dopaminergic degeneration becoming more prominent in later stages (61, 62). Cholinergic deficits have been associated with apathy, however, they do not appear to play a major role in fatigue (33, 62, 63).

To date, no imaging biomarker has been validated to distinguish between the two syndromes, and the role of fluid biomarkers in apathy remains limited (37, 64). Furthermore, most rating scales for apathy and fatigue lack domain specificity, allowing symptom overlap that can lead to misdiagnosis and introduce analytical bias, ultimately compromising the validity of research findings.

## 6 Fatigue, depression and anxiety

Fatigue is included in the Diagnostic and Statistical Manual of Mental Disorders, Fifth Edition (DSM-5) as a diagnostic criterion for depression and anxiety, both of which are common in PD (65, 66). Consequently, distinguishing fatigue as an independent symptom from these coexisting conditions can be challenging. To address these complexities, Skorvanek et al. distinguished primary fatigue (in absence of mood disorders or excessive daytime sleepiness) from secondary fatigue (co-occurs with depression, anxiety, sleep disturbances, or other factors) (22). In their study, secondary fatigue was nearly twice as common as primary fatigue and was significantly associated with older age, male gender, and higher anxiety scores. Moreover, patients with depression had increased motor and fatigue scores across all fatigue domains compared to patients with primary fatigue alone. These findings align with previous studies reporting worse motor and fatigue symptoms in depressed patients (67). However, the primary versus secondary fatigue classification underwent significant reconsideration by a panel of experts, arguing that although these neuropsychiatric symptoms are frequently comorbid with fatigue, they remain distinct and clinically dissociable phenomena (15, 20). Rather than categorizing fatigue as primary or secondary, it was proposed viewing fatigue as an intrinsic, multifactorial symptom of PD, where coexisting neuropsychiatric conditions act as modifiers, influencing its severity and presentation rather than separate etiologies. A subsequent validation of this framework in 240 patients confirmed a coherent clinical subtype valuable for both practice and research (68).

Depression is one of the most consistently documented neuropsychiatric symptoms associated with fatigue in PD (69–71). A meta-analysis confirmed significant associations between fatigue and depression in 18 out of 23 included studies, revealing that fatigued patients report more depressive symptoms and have a higher risk of receiving a depression diagnosis compared to those without fatigue (4). Increased depressive symptoms mediate the impact of PD severity on various fatigue domains (general fatigue, reduced activity and mental fatigue), underscoring the necessity of adequately managing depression within comprehensive PD care (71). Nevertheless, fatigue may persist even after successful treatment of depression (5).

Anxiety shows a similarly robust, though less explored, relationship with fatigue. Multiple studies report significantly higher anxiety scores in fatigued versus non-fatigued patients, and meta-analytic evidence confirms this association (4, 51, 52, 72–74). In *de novo* PD patients, distressing fatigue correlates with episodic anxiety independently of other NMS, suggesting shared underlying pathogenic mechanisms between these symptoms (52). Similar associations have been observed in PD patients already treated with levodopa (75, 76). Whether episodic anxiety could influence the subjective perception of fatigue, fatigue precipitates episodic anxiety, or both conditions arise from shared circuitry warrants further investigation (52). Because anxiety magnifies the overall non-motor burden and exacerbates fatigue’s negative impact on quality of life (4), systematic assessment and treatment of anxiety should be integral to any therapeutic strategy aimed at alleviating fatigue in PD.

Similar to what has been observed in fatigue, metabolic and functional imaging studies have shown that depression and anxiety in PD involve widespread abnormalities across prefrontal and corticolimbic–basal ganglia circuits, with dopaminergic and serotonergic dysfunctions playing a central role in their pathophysiology (24, 25, 54, 62, 77–79). The involvement of mesostriatal and mesolimbic dopaminergic pathways and serotonergic deficits within limbic cortical areas have been well documented in both mood disturbances (58, 61, 80–87). These findings support the hypothesized degenerative pathway in PD, where alpha-synuclein pathology originates in the olfactory bulb and progressively spreads to limbic structures, disrupting serotonergic projections and prefrontal–basal ganglia circuits, and ultimately manifesting clinically as a distinct “serotonergic” phenotype characterized by fatigue, mood disturbances, and pain (88–90). Noradrenergic deficits across limbic structures (particularly involving the locus coeruleus, thalamus, amygdala, and anterior cingulate cortex) have been identified using [^11^C]RTI-32 PET and associated with depression and anxiety, whereas neocortical cholinergic dysfunction has been primarily linked to depression in PD (58, 91).

In relation to biofluid investigations, higher levels of NfL and inflammatory markers in serum have been associated with the severity and progression of fatigue, depression and anxiety in PD, suggesting a potential biological link between these NMS (12, 37, 41, 42, 64).

The shared neurobiological substrates, involving disruptions in prefrontal, limbic, and nigrostriatal pathways involving dopaminergic, serotonergic, and noradrenergic projections, together with potential contribution of inflammatory and neurodegenerative mechanisms, may underlie the symptomatic overlap between fatigue and mood disorders in PD, making their clinical distinction more challenging.

## 7 Fatigue and cognitive impairment

Cognitive impairment in PD can manifest at any stage of the disease (92). Early deficits predominantly involve attention, working memory, and executive functioning, linked to fronto-striatal dysfunction, whereas language and memory impairments associated with posterior cortical involvement tend to manifest in more advanced stages (93). Although cognitive impairment and fatigue are regarded as independent symptoms with distinct courses, their high prevalence frequently results in their co-occurrence.

It is important to recognize that fatigue, within its multidimensional nature, also includes a cognitive component, often referred to as cognitive fatigue (3). This form of fatigue is characterized by an increased mental effort required to perform cognitive tasks, which may manifest solely as subjective complaints in some cases or result in an objective impairment in cognitive performance in others. Maladaptive metacognitive beliefs, particularly those related to low cognitive confidence, are thought to contribute to cognitive fatigue (94).

Multiple studies have linked fatigue to increased subjective cognitive complaints, reduced cognitive performance, and a higher risk of future cognitive decline in PD (11, 13, 56, 95–99). The majority of this literature indicates that fatigue-related cognitive impairment predominantly involves executive-attentional networks, suggesting a potential role of disrupted striato-thalamo-cortical connectivity with the prefrontal cortex (13, 100). Although less frequently reported, some studies have also found associations with poorer visuospatial abilities or episodic memory (both markers of poor cognitive prognosis), whereas others have failed to identify a consistent relationship between fatigue and cognitive impairment or decline (52, 67, 97, 101, 102).

Functional neuroimaging studies, alongside other biomarker-based investigations, have sought to elucidate the overlapping neural correlates and markers between fatigue and cognitive impairment in PD. Serotonergic dysfunction, which has been clearly linked to fatigue, also plays a significant role in the pathophysiology of cognitive impairment in PD. Meta-analytic data show that lower SERT binding in the caudate correlates with worse cognitive scores (103). Additionally, [11C]DASB PET findings suggest that serotonergic pathology is associated with cognitive decline in mild to moderate PD and reduced cortical serotoninergic innervation correlates with increased amyloid deposition (104). Dopaminergic denervation is also a well-known contributor to PD-associated cognitive impairment, particularly in the early stages (105). However, as discussed previously, the role of the dopaminergic system in fatigue remains less well defined. Similar to apathy, the cholinergic system is strongly implicated in cognitive decline, but does not appear to contribute substantially to fatigue (33, 62, 63). Altogether, the overlap in predominantly fronto-striatal and limbic structures, along with the degeneration of monoaminergic systems, may underlie the associations and the co-occurrence of fatigue and cognitive decline in PD.

An event-related potential (ERP) study found increased latency and amplitude of the P3a component of the P300, suggesting deficits related to fronto-striatal circuits and further supporting the link between fatigue and more pronounced cognitive impairment in PD (106). Regarding biofluid studies, elevated levels of inflammatory markers and NfL have both been associated with cognitive decline and fatigue (12, 37, 42, 107–109).

Regardless of whether fatigue contributes to cognitive deficits or cognitive demands exacerbate fatigue, it is clear that these symptoms can emerge independently and follow distinct courses (110, 111). However, their clinical and pathophysiological overlap, along with differences in their management, highlights the importance of evaluating cognition in PD patients presenting with fatigue.

## 8 Treatment of fatigue in PD

Fatigue is often underrecognized in routine care, and evidence-based treatments are limited (Table 2) (18, 112). Randomized clinical trials of pharmacologic or nonpharmacologic interventions have generally evaluated fatigue as a secondary outcome and have seldom stratified participants for baseline fatigue severity (113–116).

**Table 2:** Pharmacological and non-pharmacological treatment interventions for PD-related fatigue.

Drug/intervention	Mechanism of action	Comments
Levodopa	Precursor of dopamine	Mixed evidence; one RCT indicated improvement of physical fatigue, however, the ELLDOPA trial found no significant effect (118, 130)
LCIG	Precursor of dopamine	Low quality evidence from observational studies; improvement of NMSS sleep/fatigue domain scores (131, 132)
Rotigotine	Dopamine receptor agonist	Limited evidence from post hoc analysis of RECOVER study suggesting a positive effect on fatigue (133)
Rasagiline	MAO-B inhibition	Post-hoc analysis of ADAGIO trial and pilot RCT showed improvement of fatigue (16, 117) The only treatment considered “*possibly useful”* by the MDS-Evidence Based Medicine (18)
Safinamide	MAO-B inhibition and glutamatergic modulation	Low quality evidence from observational studies; potential benefit on fatigue (122, 123)
Modafinil	Promotes wakefulness via multiple arousal pathways	Limited evidence; may improve physical fatigability (114, 134)
Methylphenidate	Dopamine and norepinephrine reuptake inhibition	Limited evidence; may improve cognitive and physical fatigue (17)
Doxepin	Tricyclic antidepressant with selective histaminergic antagonistic action	Limited evidence; may improve fatigue (116)
Memantine	Uncompetitive, low-affinity, open-channel blocker of NMDA-type glutamate receptor	No significant benefit on fatigue (135)
Caffeine	Antagonism of adenosine-2A receptors	No significant benefit on fatigue (115)
STN-DBS	Modulation of neuronal activity in the STN	Low quality evidence from an observational study; improvement of NMSS sleep/fatigue domain scores (132)
Physical exercise	Improves neuroplasticity, energy regulation, and inflammation	Moderate evidence from meta-analysis; best non-pharmacologic strategy available (127)
tDCS	Non-invasive neuromodulation that modulates cortical excitability and network connectivity	Preliminary evidence from a pilot RCT showed significant improvement in total NMSS and sleep/fatigue domain scores (128)
CBT	Targets maladaptive thoughts; improves symptom perception and coping strategies	Limited evidence; may improve cognitive/emotional fatigue (116)
Vestibular rehabilitation	Enhances arousal and sensory integration via balance training	Limited evidence; may improve fatigue perception (136)
Acupuncture	Not known; nonspecific/placebo effect	Limited evidence; may improve fatigue but no greater benefit than sham treatments (15)

*Adapted from (112). RCT, randomized clinical trial; LCIG, levodopa-carbidopa intestinal gel infusion; NMSS, Non-Motor Symptoms Scale; MAO-B, monoamine oxidase B; MDS, Movement Disorder Society; NMDA, N-methyl-D-aspartate receptor; STN-DBS, subthalamic nucleus deep brain stimulation; tDCS, transcranial direct current stimulation; CBT, cognitive-behavioral therapy.

Among antiparkinsonian agents, the strongest, albeit still limited, evidence supports rasagiline, a selective MAO-B inhibitor that enhances dopaminergic transmission (18, 112). The latest MDS evidence review highlights the paucity of high-quality trials: although one study reported a benefit, methodological limitations led the panel to rate rasagiline as only *“possibly useful”* once secondary causes of fatigue have been excluded (18). In the ADAGIO delayed-start trial, rasagiline 1 mg and 2 mg slowed the progression of fatigue over 72 weeks versus placebo, as assessed with the PFS (117). A smaller 12-week study of 30 patients likewise showed significant improvements on the total MFI, particularly on the mental fatigue subscore (16).

Importantly, fatigue has also been reported in close association with motor fluctuations in patients receiving chronic levodopa therapy, where it may emerge as a manifestation of non-motor fluctuations. Several studies have shown that fatigue fluctuates significantly with motor states, being more severe and frequent in the “off” state compared to the “on” state (118–120). This pattern suggests that fatigue is closely linked to the hypodopaminergic state during motor “off” periods, paralleling motor symptom response and supporting the contribution of dopaminergic mechanisms to fatigue expression in at least a subset of patients. Recognition of fatigue as a non-motor fluctuation is essential for clinical management, as it opens the possibility of treatment optimization strategies such as adjusting levodopa dosing and timing or using extended-release formulations (119).

Beyond dopaminergic mechanisms, dysregulation of other neurotransmitters, particularly glutamate, appears to contribute to PD pathophysiology and its complications (121). Emerging evidence suggests that safinamide may alleviate several NMS, including fatigue (122, 123). Its favorable effects on pain and apathy have been attributed to its glutamate-modulating properties (124, 125). Nevertheless, most available data derive from post-hoc analyses of motor-fluctuation trials or from uncontrolled studies that used nonspecific fatigue scales (70, 126).

Other approaches, including acupuncture, modafinil, and methylphenidate, remain investigational (98). Among non-pharmacologic options, meta-analytic evidence indicates that structured exercise programs produce a modest but significant reduction in fatigue relative to passive or placebo control conditions, underscoring exercise as a promising strategy (127). A recent study in individuals with early-onset PD showed that transcranial direct current stimulation (tDCS) significantly reduced the total NMSS total and sleep/fatigue item scores (128). Preliminary data suggest that behavioral interventions, especially vestibular rehabilitation training, may improve PD-related fatigue (129).

Consequently, fatigue in PD remains a major unmet therapeutic need. Well-designed, double-blind clinical trials are needed to advance the evaluation of both pharmacological and non-pharmacological interventions (15, 20).

## 9 Future directions

Extensive evidence supports the hypothesis that fatigue, although an independent symptom, is often associated with other neuropsychiatric symptoms that should be carefully evaluated as part of a comprehensive, individualized assessment. Future research should consider this and focus on three mutually reinforcing objectives: (i) standardizing diagnostic criteria and measurement tools, (ii) identification of fatigue-related biomarkers, and (iii) conducting mechanism-based therapeutic trials.

### 9.1 Standardizing diagnostic criteria and measurement tools

A key obstacle in addressing PD-related fatigue is the inconsistent use of terminology and the frequent overlap with other NMS. Terms such as *cognitive fatigue*, *central fatigue*, and *mental fatigue* are often used interchangeably, underscoring the need for clearer conceptual distinctions and terminological consistency (9, 20, 137). The taxonomy proposed by Kluger et al. provides a valuable framework in this regard by explicitly separating *fatigue* (subjective perception) from *fatigability* (objective performance decline) and by calling for specification of clinical significance, causal factors, and performance domains affected (9). Applying this framework in PD research could reduce conceptual ambiguity, foster development of instruments that capture both domains, and clarify the mechanisms linking fatigue with other neuropsychiatric symptoms. Future efforts should also focus on refining conceptual definitions and adopting the standardized diagnostic criteria proposed by Kluger et al. (15, 20) in both clinical and research contexts. Moreover, developing fatigue-specific rating tools that clearly differentiate affective and cognitive dimensions is essential. Because fatigue frequently co-occurs with apathy, depression, anxiety, and emerging cognitive decline, comprehensive neuropsychological assessment should become routine to identify these symptoms and ensure their appropriate management.

### 9.2 Identification of fatigue-related biomarkers

**Figure 1:**
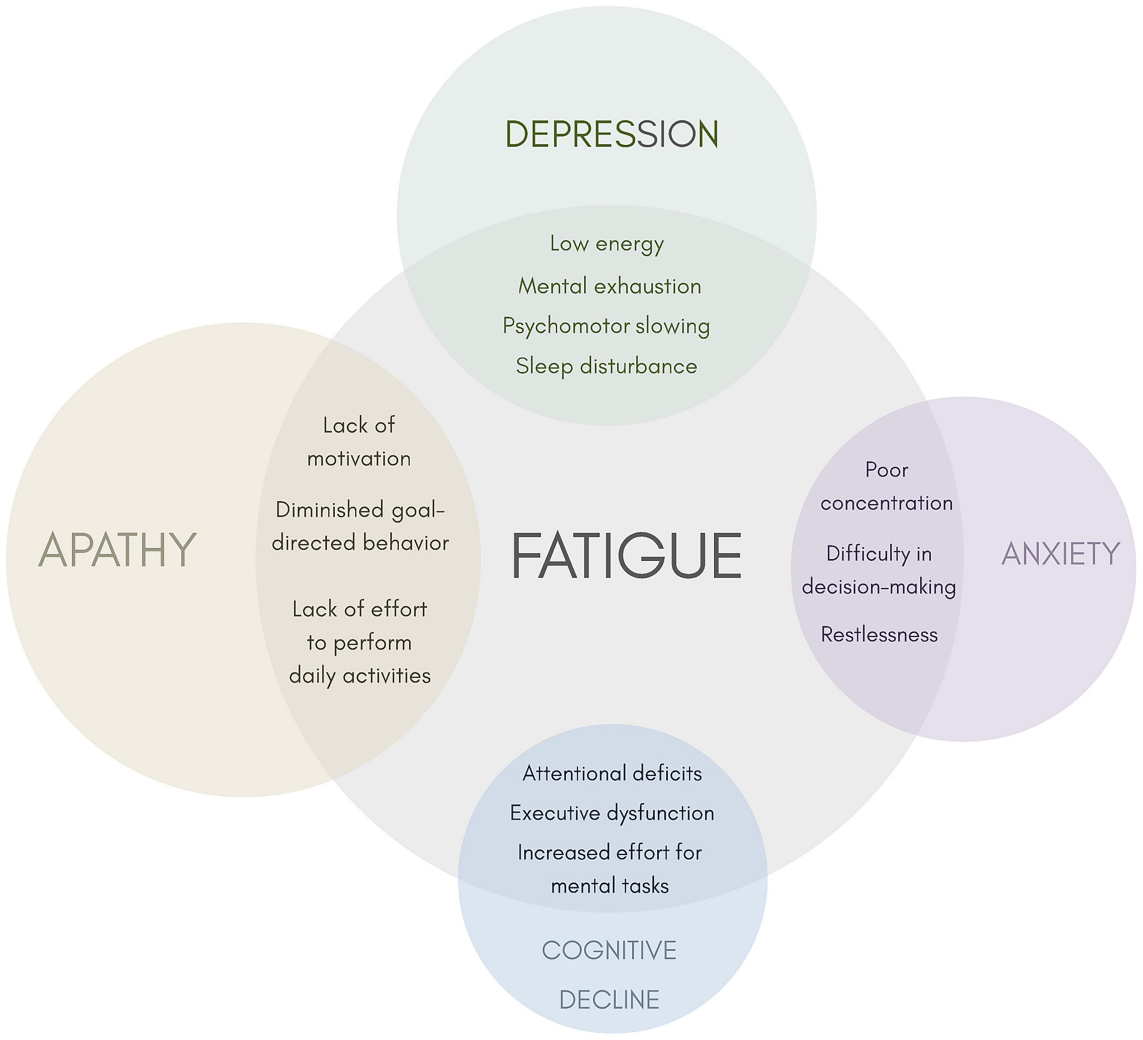
Overlapping symptoms of fatigue with apathy, depression, anxiety, and cognitive decline. The size of each circle represents the degree of overlap between fatigue and each neuropsychiatric symptom.

Fatigue in PD arises from a complex interplay of circuit dysfunction, disturbance in neurotransmitter systems, and pro-inflammatory processes. Because these abnormalities are increasingly measurable through advanced neuroimaging, electrophysiology, and fluid assays, they represent promising candidate biomarkers to improve diagnostic specificity. To better understand these mechanisms, there is a need for longitudinal cohorts that integrate standardized clinical phenotyping and neuropsychological assessment with advanced neuroimaging and fluid biomarker analysis. Such comprehensive datasets would help disentangle fatigue from overlapping neuropsychiatric symptoms (Figure 1). Figure 1 illustrates the symptomatic overlap of fatigue with apathy, depression, anxiety, and cognitive impairment, highlighting their frequent co-occurrence, which complicates clinical differential diagnosis and underscores the need for symptom-specific biomarkers.

### 9.3 Conducting mechanism-based therapeutic trials

Similar to apathy in PD, it is possible that in the future, identifying the specific cognitive or emotional symptoms associated with fatigue, or the specific neurotransmitter impairment in a given patient, may allow for targeted or combined therapeutic approaches (8, 57). Candidate biomarkers derived from neuroimaging, electrophysiology, and inflammatory assays may prove particularly valuable in this regard, enabling the stratification of patients into biologically defined subgroups and guiding mechanism-based therapeutic development. Future research should address both pharmacological and non-pharmacological strategies, with interventions tailored to the individual profiles of patients. Placebo-controlled trials must adopt the refined diagnostic criteria and include consistent clinical outcomes alongside sensitive biomarker measures (15, 20). Non-pharmacological approaches, such as structured exercise programs and cognitive-behavioral interventions, deserve further investigation, as they may target the multiple dimensions of fatigue more effectively than single-modality treatments (3, 4, 127, 129).

## 10 Conclusion

Fatigue is a highly prevalent and disabling NMS in PD, yet it remains poorly understood and frequently overlooked in clinical care. Its substantial overlap with neuropsychiatric symptoms, such as apathy, depression, anxiety, and cognitive impairment, poses a major challenge for accurate diagnosis and treatment. Disentangling fatigue from these co-occurring symptoms is essential to ensure precise clinical characterization and guide effective interventions. Despite sharing neural circuits and neurotransmitter systems with other neuropsychiatric symptoms, fatigue is a distinct syndrome that warrants separate and independent assessment. Integrating comprehensive clinical and neuropsychological evaluations with imaging and fluid biomarkers is crucial to enhance diagnostic specificity and reduce misclassification. Future efforts must prioritize the clear delineation of fatigue as a measurable and biologically grounded entity to advance treatment strategies and ultimately improve patients’ quality of life.
